# The effect of N-acetyl-l-cysteine (NAC) on liver toxicity and clinical outcome after hematopoietic stem cell transplantation

**DOI:** 10.1038/s41598-018-26033-z

**Published:** 2018-05-29

**Authors:** Ibrahim El-Serafi, Mats Remberger, Ahmed El-Serafi, Fadwa Benkessou, Wenyi Zheng, Eva Martell, Per Ljungman, Jonas Mattsson, Moustapha Hassan

**Affiliations:** 1 ECM, KFC, Department of Laboratory Medicine, Karolinska Institutet, Stockholm, Sweden; 2 Department of Clinical and Experimental Medicine, Linköping University, Linköping, Sweden; 3 Center for Allogeneic Stem Cell Transplantation, Karolinska University Hospital, Stockholm, Sweden; 4 Department of Oncology and Pathology, Karolinska Institutet, Stockholm, Sweden; 5 College of Medicine, University of Sharjah, Sharjah, UAE; 6 Experimental Cancer Medicine, Clinical Research Center, Karolinska University Hospital, Huddinge, Sweden

## Abstract

Busulphan (Bu) is a myeloablative drug used for conditioning prior to hematopoietic stem cell transplantation. Bu is predominantly metabolized through glutathione conjugation, a reaction that consumes the hepatic glutathione. N-acetyl-l-cysteine (NAC) is a glutathione precursor used in the treatment of acetaminophen hepatotoxicity. NAC does not interfere with the busulphan myeloablative effect. We investigated the effect of NAC concomitant treatment during busulphan conditioning on the liver enzymes as well as the clinical outcome. Prophylactic NAC treatment was given to 54 patients upon the start of busulphan conditioning. These patients were compared with 54 historical matched controls who did not receive NAC treatment. In patients treated with NAC, aspartate transaminase (AST), alanine transaminase (ALT) and alkaline phosphatase (ALP) were significantly (*P* < 0.05) decreased after conditioning compared to their start values. Within the NAC-group, liver enzymes were normalized in those patients (30%) who had significantly high start values. No significant decrease in enzyme levels was observed in the control group. Furthermore, NAC affected neither Bu kinetics nor clinical outcome (sinusoidal obstruction syndrome incidence, graft-versus-host disease and/or graft failure). In conclusion: NAC is a potential prophylactic treatment for hepatotoxicity during busulphan conditioning. NAC therapy did not alter busulphan kinetics or affect clinical outcome.

## Introduction

Hematopoietic stem cell transplantation (HSCT) is a curative treatment for malignancies such as leukemia, lymphomas and some solid tumors, as well as non-malignant diseases such as metabolic disorders and aplastic anemia^1^. Several acute and chronic complications may occur and hamper treatment success, including graft-versus-host disease (GVHD), infections and sinusoidal obstruction syndrome (SOS, previously called venoocclusive disease, VOD).

The conditioning regimen is an important step during HSCT. Conditioning regimens contains either total body irradiation (TBI)-based (TBI and cytostatics) or chemotherapy-based (combinations of cytostatics without TBI). According to the administered doses (intensity), conditioning regimens can also be divided into myeloablative and non-myeloablative regimen^2^.

Busulphan (Bu) is chemotherapeutic agent that has been in clinical use since 1952^3^. Busulphan is predominantly metabolized in the liver by conjugation with glutathione (GSH)^4–7^. Cytosolic glutathione S-transferases (GSTs) have been identified as the enzymes catalyzing this conjugation. GSTA1 is the most active glutathione transferase in catalyzing Bu-GSH conjugation, while GSTM1 and GSTP1 are less active^8,9^. This conjugation results in the formation of sulfonium ion that is an unstable intermediate and is degraded to tetrahydrothiophene (THT). Oxidation products of THT, such as THT 1-oxide, sulfolane and 3-hydroxy sulfolane represent the majority of identified Bu metabolites^4–7^.

Busulphan was previously reported to cause liver toxicity such as SOS in patients undergoing HSCT^10–13^. SOS is an early complication and the most common causes of death in these patients^10,11^. SOS was defined by McDonlad *et al*. as the onset of two of the following occurring before day 30 post-HSCT: (1) jaundice (bilirubin > 27 µmol/L), (2) tender hepatomegaly and (3) ascites or weight gain. While Jones *et al*. narrowed the definition to the following: onset before day 21 post-HSCT of hyperbilirubinemia (bilirubin > 34 µmol/L) as well as two of the following: (1) hepatomegaly, (2) ascites and (3) weight gain^14,15^.

Busulphan was reported to consume up to 60% of the hepatic GSH *in vivo* and 50% *in vitro*, thus; cells lacking GSH are more sensitive to Bu toxicity^16^. GSH is an important intracellular antioxidant and its exhaustion by cytotoxic drugs and/or radiation may contribute to the development of SOS. Moreover, endothelial cell damage due to the toxicity of conditioning regimen may lead to activation of several clotting factors^13,17^.

N-acetyl-l-cysteine (NAC) is a GSH precursor that increases the cellular content of GSH^18^. NAC is used in the treatment of hepatotoxicity caused by acetaminophen as well as for the treatment of SOS^19,20^. We have reported the use of NAC in the treatment of three patients who developed SOS after HSCT. Later, the liver enzymes of these patients decreased and all three patients achieved normal bilirubin levels and prothrombin times^20^. We have also reported that NAC does not interfere with the myeloablative effect of Bu *in vitro* or in animal models, and that it can be considered for SOS prophylaxis during Bu conditioning^21^. Later, these findings have been confirmed in ten patients at high risk for SOS due to pre-transplant liver disorders or elevated liver enzymes. NAC did not affect Bu kinetics or its myeloablative effect. Moreover, liver enzymes were normalized in all patients and none of them developed SOS^22^. Currently, all patients treated with Bu are receiving prophylactic NAC upon the start of Bu conditioning according to the hospital protocol.

In this study, we investigated the effect of prophylactic treatment of NAC during Bu conditioning on the clinical outcome in terms of liver enzymes, relapse, SOS, GVHD and graft rejection.

## Results

### Liver enzymes

**Figure 1:**
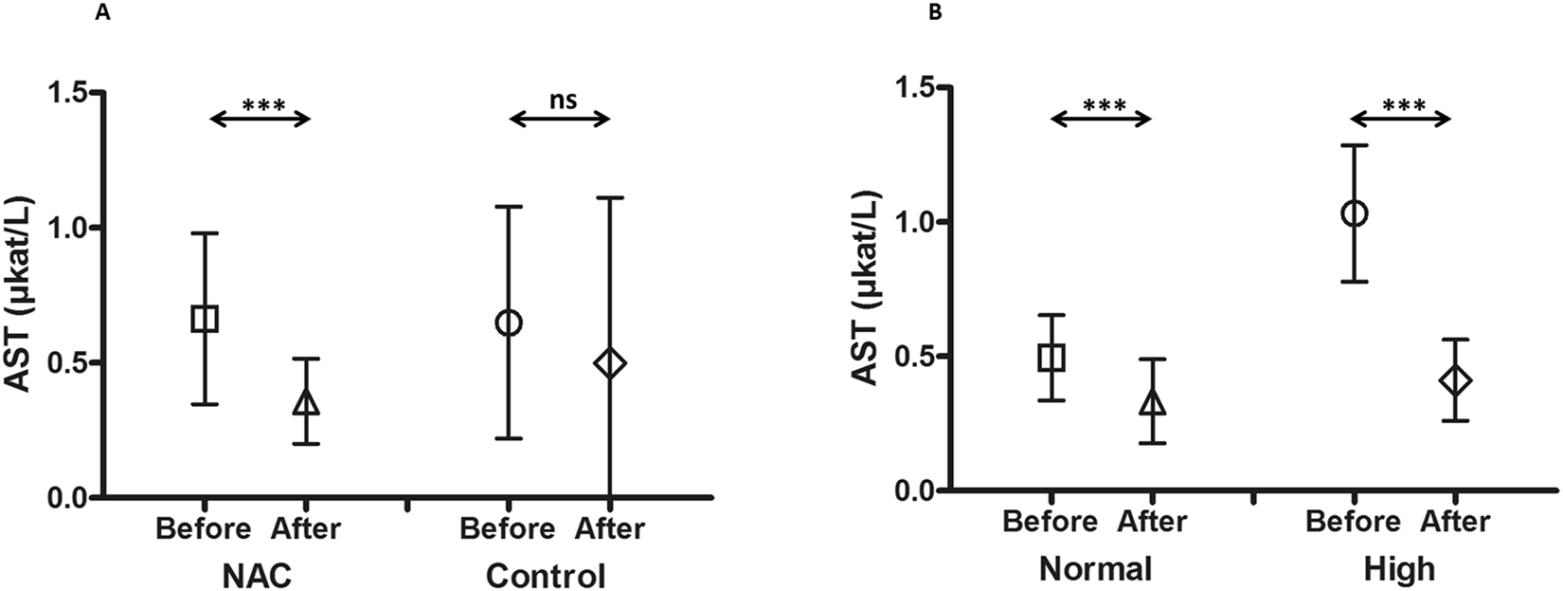
The mean aspartate transaminase (AST) values before and after busulphan conditioning in patients treated with N-acetyl-l-cysteine (NAC) versus the control group. In patients treated with NAC (n = 54), AST was significantly (*P* < 0.0001) decreased after conditioning compared to the values before the start of conditioning. AST mean value was 0.36 ± 0.16 µkat/L after conditioning compared to 0.66 ± 0.32 µkat/L before the start of conditioning (**A**). On the other hand, there was no significant decrease for AST in the control group (*P* = 0.15, n = 54). The mean values for AST before and after conditioning were 0.65 ± 0.43 µkat/L and 0.50 ± 0.61 µkat/L, respectively (**A**). Patients treated with NAC were further divided into two subgroups; the first subgroup included patients with normal AST before the start of conditioning (the mean value was 0.49 ± 0.16 µkat/L, n = 37), while the other group had high AST before the start of the conditioning (1.03 ± 0.25 µkat/L, n = 17). After conditioning, there was a significant decrease in AST for those patients who had high levels (mean = 0.41 ± 0.15 µkat/L, *P* < 0.0001) (**B**). The mean values for patients with normal AST did not increase, but further decreased (AST mean = 0.33 ± 0.16 µkat/L, *P* < 0.0001) (**B**). Symbols present both mean and standard deviation.

**Figure 2:**
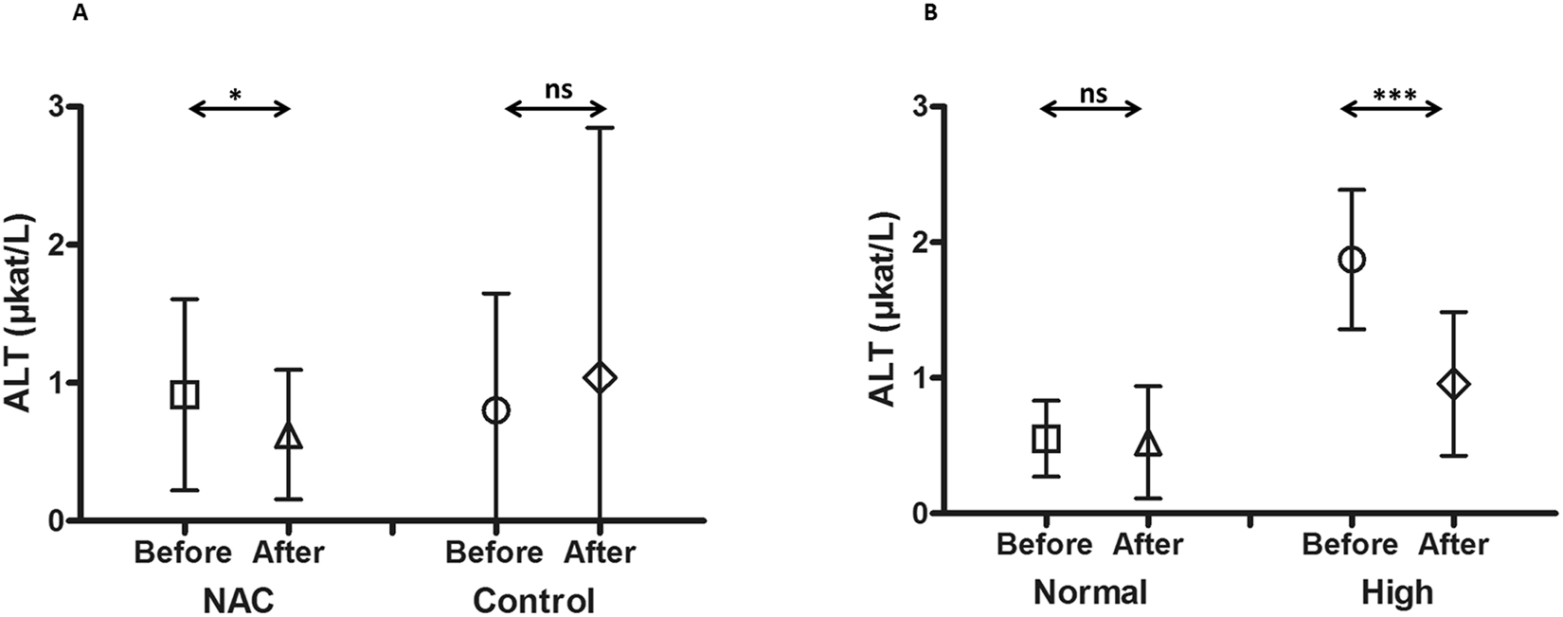
The mean alanine transaminase (ALT) values before and after busulphan conditioning for patients treated with N-acetyl-l-cysteine (NAC) versus the control group. In patients treated with NAC (n = 54), ALT was significantly (*P* = 0.016) decreased after conditioning compared to the values before the start of conditioning. ALT mean value was 0.62 ± 0.47 µkat/L after conditioning compared to 0.91 ± 0.69 µkat/L before the start of conditioning (**A**). On the other hand, there was no significant decrease for the same enzyme in the control group (*P* = 0.39, n = 54). The mean values for ALT before and after conditioning were 0.80 ± 0.85 µkat/L and 1.04 ± 1.81 µkat/L, respectively (**A**). Patients treated with NAC were further divided into two subgroups; the first subgroup included patients with normal ALT before the start of conditioning (the mean value was 0.55 ± 0.28 µkat/L, n = 37), while the other group had high ALT before the start of conditioning (1.87 ± 0.51 µkat/L, n = 17). After conditioning, there was a significant decrease in ALT for those patients who had high levels (mean = 0.96 ± 0.53 µkat/L, *P* < 0.0001) (**B**). The mean values for patients with normal ALT did not increase but further decreased (ALT mean = 0.53 ± 0.41 µkat/L, *P* = 0.75) (**B**). Symbols present both mean and standard deviation.

**Figure 3:**
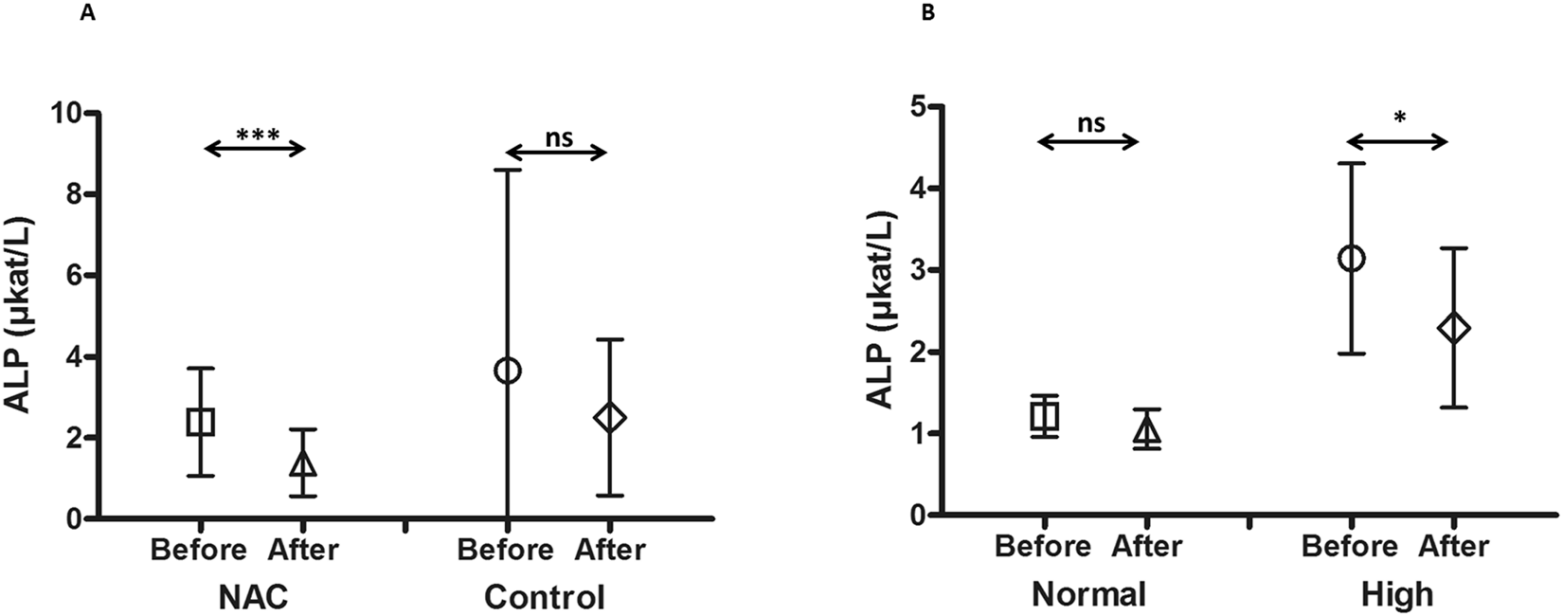
The mean alkaline phosphatase (ALP) values before and after busulphan conditioning for patients treated with N-acetyl-l-cysteine (NAC) versus the control group. In patients treated with NAC (n = 54), ALP was significantly (*P* = 0.0002) decreased after conditioning compared to the values before the start of conditioning. ALP mean value was 1.39 ± 0.82 µkat/L after conditioning compared to 2.39 ± 1.32 µkat/L before the start of conditioning (**A**). On the other hand, there was no significant decrease for the same enzyme in the control group (*P* = 0.12, n = 54). The mean values for ALP before and after conditioning were 3.67 ± 4.94 µkat/L and 2.50 ± 1.92 µkat/L, respectively (**A**). Patients treated with NAC were further divided into two subgroups; the first subgroup involved patients with normal ALP before the start of conditioning (the mean value was 1.21 ± 0.25 µkat/L, n = 37), while the other group had high ALP before the start of conditioning (3.14 ± 1.16 µkat/L, n = 17). After conditioning, there was a significant decrease in ALP for those patients who had high levels (mean = 2.29 ± 0.97 µkat/L, *P* = 0.046) (**B**). The mean values for patients with normal ALP did not increase but further decreased (ALP mean = 1.06 ± 0.24 µkat/L, *P* = 0.19)(**B**). Symbols present both mean and standard deviation.

In patients treated with NAC, aspartate transaminase (AST), alanine transaminase (ALT) and alkaline phosphatase (ALP) were significantly (*P* < 0.0001, *P* = 0.016 and *P* = 0.0002 respectively, t-test) decreased after conditioning compared to their start values before the initiation of conditioning. AST mean value was 0.36 ± 0.16 µkat/L after conditioning compared to 0.66 ± 0.32 µkat/L before the start of conditioning (Fig. 1A). ALT mean value was 0.62 ± 0.47 µkat/L after conditioning compared to 0.91 ± 0.69 µkat/L before the beginning of conditioning (Fig. 2A). Finally, ALP mean value was 1.39 ± 0.82 µkat/L after conditioning compared to 2.39 ± 1.32 µkat/L before the initiation of conditioning (Fig. 3A).

On the other hand, no significant decrease of these enzymes levels was observed in the control group (*P* = 0.15, *P* = 0.39 and *P* = 0.12, t-test) for AST, ALT and ALP, respectively. The mean values for AST before and after conditioning were 0.65 ± 0.43 µkat/L and 0.50 ± 0.61 µkat/L, respectively (Fig. 1A), ALT before and after conditioning were 0.80 ± 0.85 µkat/L and 1.04 ± 1.81 µkat/L, respectively (Fig. 2A), and ALP before and after conditioning were 3.67 ± 4.94 µkat/L and 2.50 ± 1.92 µkat/L, respectively (Fig. 3A).

Patients treated with NAC were further divided into two subgroups. The first subgroup involved patients with normal liver status before the start of the conditioning (n = 37 patients). Their mean values were 0.49 ± 0.16µkat/L, 0.55 ± 0.28 µkat/L and 1.21 ± 0.25 µkat/L for AST, ALT and ALP respectively. The second group had high liver values before the start of the conditioning (1.03 ± 0.25 µkat/L, 1.87 ± 0.51 µkat/L and 3.14 ± 1.16 µkat/L for AST, ALT and ALP, respectively, n = 17 patients).

A significant decrease in the liver values (AST mean = 0.41 ± 0.15 µkat/L, *P* < 0.0001 (Fig. 1B), ALT mean = 0.96 ± 0.53 µkat/L, *P* < 0.0001 (Fig. 2B), ALP mean = 2.29 ± 0.97 µkat/L, *P* = 0.046, t-test (Fig. 3B)) was observed after conditioning in patients who had high liver enzymes before treatment started. The mean values for AST, ALT and ALP in patients with normal liver status did not increase but further decreased (AST mean = 0.33 ± 0.16 µkat/L, *P* < 0.0001 (Fig. 1B), ALT mean = 0.53 ± 0.41 µkat/L, *P* = 0.75 (Fig. 2B), ALP mean = 1.06 ± 0.24 µkat/L, *P* = 0.19, t-test (Fig. 3B)). Patients in the control group were also divided into two subgroups according to their liver status before the start of the conditioning and presented in the Supplementary Fig. S1. In addition, the individual values for each patient are presented in the Supplementary Figs S2–S6.

### Clinical outcome

**Figure 4:**
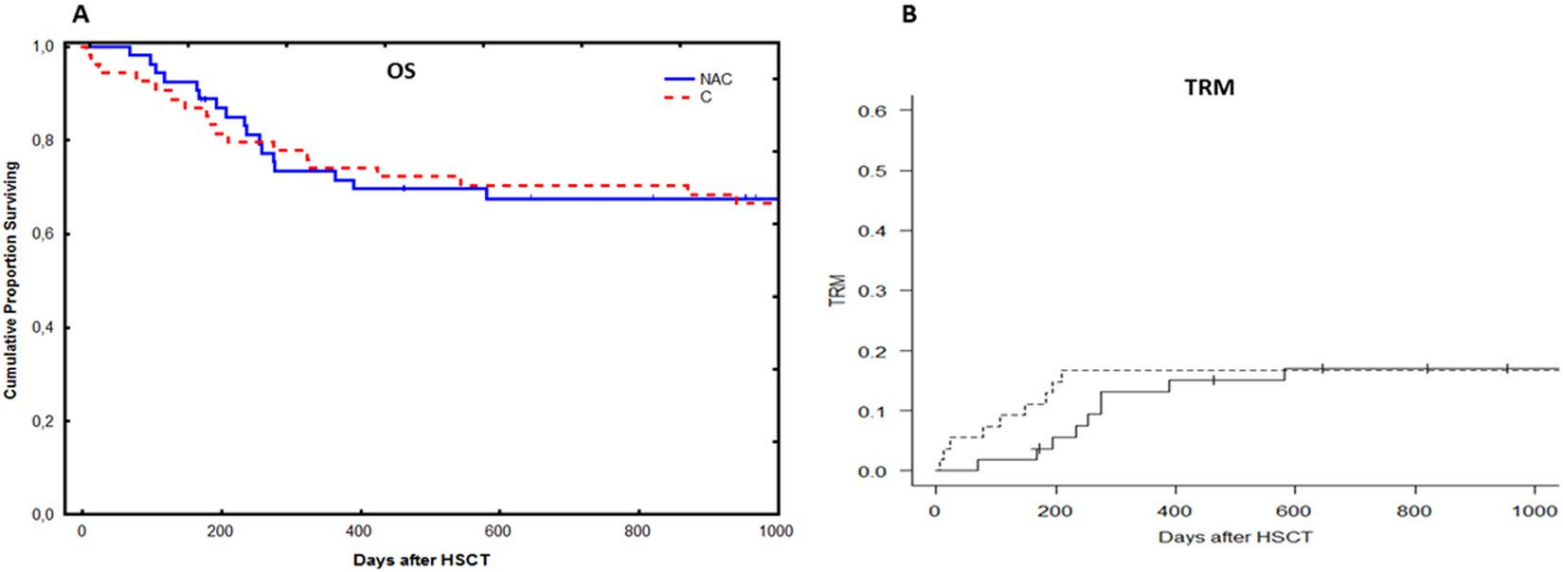
The distribution of survival and transplant-related mortality for patients treated with N-acetyl-l-cysteine (NAC) versus the control group. For both (**A**) overall survival (OS) and (**B**) transplant-related mortality (TRM), no significant difference was observed between the two groups of patients. Some of the patients treated with NAC were transplanted recently, so we were unable to follow their survival for more than one year. 4 patients are still within the first year of survival after transplantation. Solid line = NAC, dashed line = Controls.

**Figure 5:**
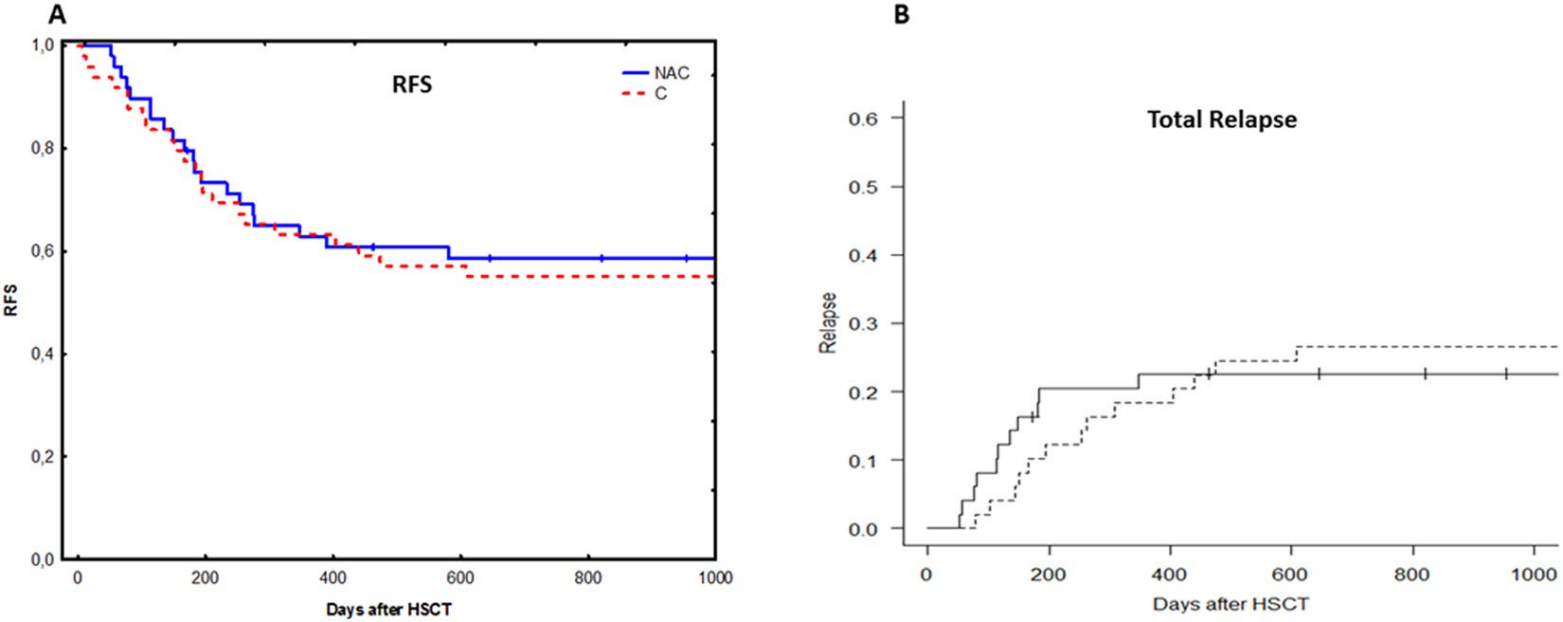
The distribution of relapse for patients treated with N-acetyl-l-cysteine (NAC) versus the control group. For both (**A**) relapse free survival (RFS) and (**B**) total relapse, no significant difference was observed between the two groups of patients. Solid line = NAC, dashed line = Controls.

**Figure 6:**
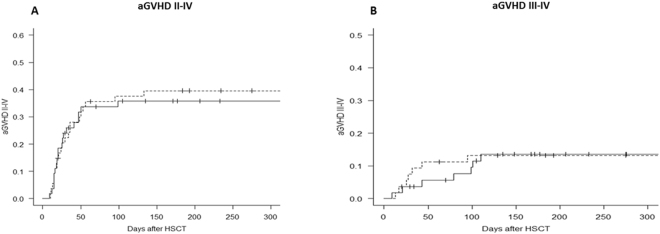
The distribution of acute graft versus host disease (aGVHD) for patients treated with N-acetyl-l-cysteine (NAC) versus the control group. No significant difference was observed between the two groups of patients for aGVHD, both (**A**) grades II-IV and (**B**) grades III-IV. Solid line = NAC, dashed line = Controls.

- No significant difference was observed in Bu exposure as expressed as area under the concentration-time curve (AUC) in both NAC and control groups. The mean Bu AUC after the first dose was 10588 ng.hr/L compared to 9324 ng.hr/L in the control group. Bu AUCs have been calculated over the 4 days of treatment in NAC group. No significant difference was observed through the conditioning period. The mean Bu AUCs were 10588, 11008, 11813 and 11000 ng.hr/L from day 1 to day 4.
- No significant difference was observed between the two groups in regard to clinical outcome such as SOS, acute or chronic GVHD, relapse, graft failure and survival (Figs 4, 5, and 6). The survival was followed up for three years in the control group while in NAC group the follow up time was three years in 50 out of 54 patients and over 100 days in 4 patients.

**Figure 7:**
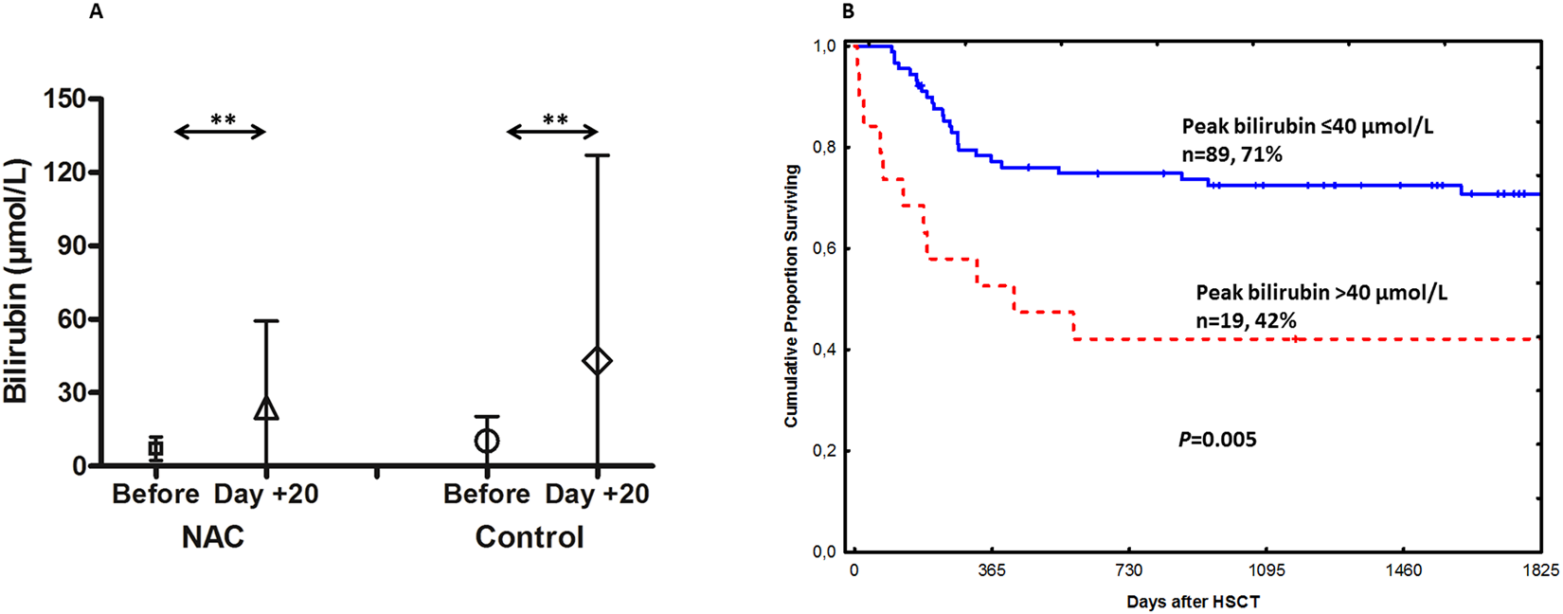
The mean bilirubin values in control and NAC treated patients (**A**). The effect of peak bilirubin at day +20 on patients’ survival (**B**). Peak bilirubin at day +20 was measured as an evidence for the clinical outcome (survival). (**A**) Bilirubin values at day +20 were highly elevated in the control group compared to NAC group; in patients treated with NAC, the mean value for bilirubin at day +20 was 24.2 ± 36.0 µmol/L compared to 7.2 ± 4.9 µmol/L before the start of conditioning (*P* = 0.001, t test). The mean value for bilirubin at day +20 in the control group was 42.4 ± 85.4 µmol/L compared to 10.4 ± 10.1 µmol/L before the start of conditioning (*P* = 0.005, t-test). Symbols present both mean and standard deviation. (**B**) Patients with bilirubin > 40 µmol/L (n = 19, 4 patients from NAC group and 15 patients from control group) had significantly lower survival compared to patients with bilirubin ≤ 40 µmol/L, *P* = 0.005. Solid line = peak bilirubin ≤ 40 µmol/L, dashed line = peak bilirubin > 40 µmol/L.

Bilirubin values at day +20 were highly elevated in the control group compared to NAC group; in patients treated with NAC, the mean value for bilirubin at day +20 was 24.2 ± 36.0 µmol/L compared to 7.2 ± 4.9 µmol/L before the start of conditioning (*P* = 0.001, t-test). On the other hand, the mean value for bilirubin at day +20 in the control group was 42.4 ± 85.4 µmol/L compared to 10.4 ± 10.1 µmol/L before the start of conditioning (*P* = 0.005, t-test) (Fig. 7A). At day +20, there were only 4 patients with bilirubin > 40 µmol/L in NAC group compared to 15 patients in the control group (*P* = 0.01).

Patients with bilirubin > 40 µmol/L (n = 19, 4 patients from NAC group and 15 patients from control group) at day +20 showed significantly lower survival compared to patients with bilirubin ≤ 40 µmol/L, *P* = 0.005 (Fig. 7B).

Relapse was relatively higher in the control group (16 patients compared to 13 patients treated with NAC). Interestingly, none of the patients treated with NAC developed any molecular relapse compared to 5 patients in the control group (Table 1).

**Table 1:** Patient characteristics and clinical outcome.

	Patients treated with NAC	Control group without NAC
***Patients’ characteristics***		
No of patients	54	54
Sex (M/F)	32/22	32/22
***Diagnosis***		
AML	36	33
ALL	1	1
MDS	9	12
CML	3	3
Non-malignant disease	5	5
Age, mean (range)	27 (1–59)	28 (<1–58)
Children (<18 years)	22 (41%)	19 (35%)
***Donor***		
Sibling	11	11
MUD	37	38
Mismatch	6	5
***GVHD prophylaxis***		
CsA + MTX	50	47
CsA + Prednisolone	3	3
Tacrolimus + Sirolimus	1	4
***Stem cell source***		
BM/PBSC/CB	16/35/3	15/36/3
***Clinical outcome***		
SOS	3 (5.5%)	3 (5.5%)
Any aGVHD	31 (57%)	31 (57%)
Any cGVHD	14 (27%)	18 (36%)
GF	3 (5.5%)	4 (7.4%)
Relapse	13 (24%)	16 (30%)
(hematological/molecular)	(13/0)	(11/5)
Survival > 1000 days	52/54 (96.3%)	50/54 (92.6%)
No./Total > 1 year	38/50 (76%)	40/54 (74%)
Bilirubin > 40 µmol/L	4/22 (18.2%)	15/22 (68.2%)

Abbreviations: AML; acute myeloid leukemia, ALL; acute lymphoid leukemia, BM; bone marrow, CB; cord blood, CML; chronic myeloid leukemia, CsA; cyclosporine F; female, aGVHD; acute graft-versus-host disease, .cGVHD; chronic graft-versus-host disease. GF; graft failure, M; male, MDS; myelodysplastic syndrome, MTX; methotrexate, MUD; matched unrelated donor, NAC; N-acetyl-l-cysteine, PBSC; peripheral blood stem cell, SOS; Sinusoidal obstruction syndrome.

## Discussion

HSCT is a curative treatment for several malignant and non-malignant diseases. However, the results are far from satisfactory due to life threatening toxicities and adverse-effects. These toxicities are mostly due to the conditioning regimen used prior to HSCT and several strategies have been introduced to improve the clinical outcome^23–31^.

Busulphan is mainly used as a myeloablative agent and as an alternative to TBI^32^. Several serious complications such as elevated liver enzymes, SOS, hemorrhagic cystitis, interstitial pneumonia and mucositis have been correlated to high-dose busulphan^10–12,15,33^. Busulphan is known to have a narrow therapeutic window and its adverse effects are delayed^34,35^.

As mentioned earlier, busulphan is conjugated to GSH via GSTs, mainly GSTA1^9^. Different GSTA1 polymorphisms in addition to the involvement of other isoforms such as GSTP1 and GSTM1 were reported to affect Bu kinetics^8,9,36^. NAC is known to increase the cellular content of GSH^18^ and it is used in the treatment of SOS and hepatotoxicity caused by acetaminophen^19,20^. Moreover, it was reported that NAC increases liver blood flow and improves liver function in septic shock patients^37^.

In our present study, we investigated the role of NAC as prophylactic treatment during Bu therapy in reducing liver toxicity if NAC treatment started at the beginning of conditioning, not after the elevation of liver enzymes. Our group has previously reported that NAC does not interfere with the myeloablative effect or kinetics of Bu and can be used as SOS prophylactic during Bu treatment^21,22^.

In patients treated with NAC, AST, ALT and ALP were significantly decreased after conditioning compared to their start values. No significant decrease in these enzymes was found in the control group (after Bu treatment). However; ALT, which is known to be more specific for liver injury^38^ was further elevated by the end of conditioning. The reduction in the mean values for AST and ALP in the control group can be due to the administration of the liver prophylactic drug, ursodeoxycholic acid^39,40^. Moreover, the standard deviations for all enzymes after Bu treatment were higher in the control group compared to that seen in patients received prophylactic NAC treatment. One possible explanation is that NAC may have an additive effect that significantly caused a decrease in the liver enzymes and improved its function.

Patients treated with NAC were further divided into two subgroups according to their liver status before Bu conditioning. Interestingly, for patients with high liver values AST, ALT and ALP were significantly decreased or normalized by the end of conditioning. Additionally, the mean values for patients with normal liver status before conditioning did not increase, but further decreased.

To the best of our knowledge, this is the first study containing relatively large number of well-matched group of patients to report a significant reduction in liver enzymes using NAC as prophylactic treatment during high-dose Bu-treatment. Our group has previously reported normalized liver values in ten patients received concomitant NAC treatment along with Bu conditioning^22^.

De Leve *et al*. showed that treatment with Bu depleted GSH, while increased levels of GSH in hepatocytes is protective against Bu toxicity^16^. GSH is exhausted due to the effect of cytostatics or radiation as reported previously and may contribute to SOS development^41^. Animals treated with NAC were protected against SOS induced by GSH exhaustion by monocrotaline^42^. On the other hand, depletion of GSH increases the toxicity of alkylating agents both *in vivo* and *in vitro*^43^. In the present study, there was no significant difference in SOS in both groups that can be due to Bu dose adjustment; however, liver toxicity, expressed as elevated liver enzymes, was much lower in patients treated with NAC.

In a pilot study, NAC showed positive effects on liver status in three patients who developed SOS after HSCT. In all the three patients, liver enzymes decreased and bilirubin levels and prothrombin times were normalized^20^. On the other hand, in a randomized study from our hospital, it has been shown that NAC did not improve liver toxicity after HSCT. The negative findings are most probably due to the fact that NAC treatment was started at the first sign of liver toxicity. Unfortunately, neither a group for prophylactic treatment with NAC nor a control group was included in the study^44^. In the present study, we started prophylactic treatment with NAC for all patients upon the beginning of conditioning regardless of their liver status. In agreement with the previous study, NAC did not affect SOS incidence^44^.

It has been previously reported that the occurrence of SOS is correlated with an increased bilirubin >27 µmol/L before day 30^14^ or >34 µmol/L at day 21^15,45,46^. Our investigation showed that only four patients with bilirubin > 40 µmol/L were observed in NAC group compared to 15 patients found in the control group. The present results confirm that NAC has significantly improved the liver functions during high dose busulphan treatment compared to the control group and hence NAC treatment may reduce the risk for SOS development. This hopefully can improve the clinical outcome, however; further long-term follow up studies are urgently warranted to confirm these findings.

Furthermore, the present results are in agreement with several previous studies showing that patients who had bilirubin levels of >40 µmol/L at day +20 showed significantly lower survival rate compared to these patients who had bilirubin < 40 µmol/L^45,47^.

Defibrotide is a deoxyribonucleic acid derivative that is used as an anticoagulant for the prophylaxis and treatment of SOS and which was approved by the FDA in April 2016 for the treatment of SOS followed HSCT^48^. Several studies have reported the importance of defibrotide as a prophylactic treatment for SOS. However, this drug is rather expensive. Veenstra *et al*., have reported that the budget impact of defibrotide for a transplantation center is relatively modest compared to the overall cost of transplantation and that it provided an important survival advantage for SOS with multi-organ dysfunction patients, which makes it cost-effective^49^. On the other hand, another study has concluded that prophylactic treatment with defibrotide for children at risk for SOS is not cost-effective with respect to transplantation-related mortality and length of hospital stay^50^. According to both McDonalds and Jones criteria, SOS is correlated to significant increase in bilirubin. In the present study, we have shown that NAC treatment has significantly reduced the liver enzymes levels as well as the bilirubin levels at day +20, thus we suggest that NAC can be a well-affordable potential prophylaxis of SOS if given concomitantly during Bu conditioning before the start of liver toxicity. However; further studies are warranted to confirm these findings.

Moreover, SOS was reported to be affected by Bu dose adjustment and therapeutic drug monitoring (TDM). The incidence of SOS was decreased from 24.1% to 3.4% in patients when dose individualization was introduced^28^. Copelan *et al*., found a significant positive correlation between high Bu AUC and SOS and early mortality; however, long-term outcome of the patients was not significantly influenced by Bu levels^51^. In our study, Bu dose adjustment for all patients may explain the low incidence of SOS observed in both groups.

Our present results showed that NAC did not affect Bu-kinetics since there was no significant difference in the mean AUC of Bu in patients treated with NAC compared to the control group. Furthermore, the Bu mean AUCs were almost constant over the 4 days of conditioning. Our current results are in good agreement with the previously reported results showing that NAC did not affect the pharmacokinetics or myelosuppressive properties of Bu^21,22^.

Busulphan administration is known to be followed by several acute and late complications after HSCT, such as GVHD, graft failure and relapse^52–55^. In our study, some of the patients treated with NAC were transplanted recently, so we were unable to follow the long term survival or late complications. However, for the rest of the patients, NAC did not affect the clinical outcome and no significant difference was observed between the two groups. Karlsson *et al*., have reported that NAC can increase acute GVHD in transplanted patients^56^. However, in their study, NAC was administered for a median of 15 days (up to 89 days in some patients) and started after transplantation, with early liver toxicity. There was no patient data, such as age, sex or diagnosis, in their report^56^.

Busulphan/cyclophosphamide (Cy) is one of the most common conditioning regimens used in the clinical setting. Cyclophosphamide is a prodrug that is converted to its active metabolite, 4-hydroxy cyclophosphamide (4-OH-Cy), through cytochrome P-450. GSH is an important enzyme for 4-OH-Cy detoxification^57,58^. Possible accumulation of the cytotoxic 4-OH-Cy due to GSH consumption by Bu may cause hepatic damage and increase the incidence of SOS^29^. The time interval between the last Bu dose and the first Cy-dose is important for the development of SOS. A significant lower incidence of SOS was found when time interval was >24 hours compared to that seen when the interval was 12 hours^29^. Moreover, alteration of the administration order of Bu-Cy to Cy-Bu gives the same engraftment outcome but reduces the toxicity of the conditioning regimen both in patients and mice^59,60^. Additionally, at steady state concentration (Css) of 800–900 ng/mL, none of the patients with myelofibrosis experienced SOS when Cy was administered prior to Bu compared to 30% in patients conditioned with Bu followed by Cy^61^.

In conclusion, N-acetylcysteine is a potential prophylactic treatment against the hepatotoxicity during busulphan-based conditioning regimens. NAC therapy did not affect the clinical outcome in terms of SOS, GVHD, relapse rate, survival rate, etc. NAC therapy is safe and does not alter busulphan pharmacokinetics or pharmacodynamics as we reported previously. The present results can be utilized for personalized medicine which in turn may affect treatment related toxicity and hence improve clinical outcome in HSCT patients.

## Patients and Methods

One hundred and eight patients were included for this study. All patients were undergoing HSCT at the Center for Allogeneic Stem Cell Transplantation (CAST), Karolinska University Hospital, Huddinge, Sweden. The study was approved by the ethical committee of Karolinska Institutet (616/03) and informed consent was obtained from the patients(or their parents). All methods were carried out in accordance with the guidelines and regulations of Karolinska University Hospital.

Patients were divided into two groups; each group consisted of 54 patients (32 males and 22 females in each group). The first group of patients received NAC twice daily at a dose of 100 mg/kg upon the start of Bu conditioning during the period of 2011–2016. The second historical group (control group) did not receive any NAC during busulphan conditioning and were being treated between 2000 and 2010 before NAC was introduced as a part of the treatment protocol. The control group was matched for the most important factors, such as sex, age, diagnosis, donor, stem cell source and GVHD prophylaxis. All patients received the same supportive care in both groups. Furthermore all patients received ursodeoxycholic acid as prophylactic treatment against liver toxicity^39,40^. SOS was diagnosed according to McDonlad criteria^14^. Patient characteristics and clinical outcome have been collected and presented in Table 1.

Busulphan was administered orally at a dose of 2 mg/kg B.I.D for 4 consecutive days^30^. Twenty four hours after the last dose of Bu, Cy was administered as i.v. infusion (60 mg/kg/day) once daily for two days to all patients. Blood samples were collected at several time points during Bu conditioning for TDM in order to perform dose adjustment. All patients received myeloablative treatment with a target Bu AUC of 9.000–12.000 ng.hr/L per dose^30^. The level of Bu in plasma was determined using gas chromatography (GC) after drug extraction, according to our previously published protocol^30^.

Liver enzymes such as AST, ALT and ALP are routinely measured before and after Bu conditioning. According to the hospital protocol, liver enzymes are evaluated in the morning (8 am) one day before the start of Bu conditioning and on day 5 morning (8 am, 24 h after Bu last dose) and the normal liver values are as follows: AST < 0.76 µkat/L, ALT < 1.1 µkat/L, ALP < 1.9 µkat/L. Additionally, to confirm the effect of busulphan treatment on the liver status, the peak bilirubin at day +20 was measured as an evidence for the clinical outcome^45–47^.

GVHD prophylaxis consisted mainly of cyclosporine (CsA) in combination with four doses of methotrexate (MTX)^62^. During the first month, blood CsA levels were maintained at 100 ng/mL for patients transplanted from sibling donors and at 200–300 ng/mL for patients transplanted from matched unrelated donor (MUD). In the absence of GVHD, CsA was successively discontinued in patients with sibling donors after three to four months and in patients with MUD after six months. Patients receiving a cord blood graft received CsA and prednisolone (n = 6). Five patients were given tacrolimus and sirolimus^63^.

Most patients received peripheral-blood stem cells (PBSC) (n = 71), while 31 received bone marrow and six patients received a cord blood graft. Before aphaeresis, all donors of PBSC were treated subcutaneously once a day with G-CSF (Rhône-Poulenc Rorer, Lyon, France or Amgen-Roche Inc., Thousand Oaks, CA, USA), 10 μg/kg/day for 4 to 6 days^63^.

Acute and chronic GVHD were diagnosed on the basis of clinical symptoms and/or biopsies (skin, liver, gastrointestinal tract, or oral mucosa) according to standard criteria^64^. The patients were treated for grade I acute GVHD with topical steroids, while higher grades of acute GVHD were treated with prednisone, starting at a dosage of 2 mg/kg/day, which was successively lowered after the initial response. Chronic GVHD was initially treated with CsA and steroids. In most cases, daily prednisone at 1 mg/kg per day and daily CsA at 10 mg/kg per day were used^65^.

Data was normally distributed and all statistical analyses and graphs were carried out using GraphPad Prism (version 4.0, GraphPad Software, Inc.) and SigmaPlot (version 12.5, Systat software, Inc.). Busulphan kinetics parameters including AUC were calculated using one-compartment open model (WinNonLin, version 2.0). The level of probability (P) for t-test is deemed to constitute the threshold for statistical significance when *P* < 0.05.

### Data availability

The datasets generated during and/or analysed during the current study are available from the corresponding author on reasonable request.

## Electronic supplementary material

Supplementary Dataset 1
