# Supplementary material for: The effect of N-acetyl-l-cysteine (NAC) on liver toxicity and clinical outcome after hematopoietic stem cell transplantation
Source: Sci Rep. 2018 May 29;8:8293. doi: 10.1038/s41598-018-26033-z (PMC5974141; doi:10.1038/s41598-018-26033-z)
Supplement: Supplementary file 1 — Supplementary Dataset 1 [file 41598_2018_26033_MOESM1_ESM.docx]

**The effect of N-acetyl-l-cysteine (NAC) on liver toxicity and clinical outcome after hematopoietic stem cell transplantation**

Ibrahim El-Serafi, MD, PhD^1,2^, Mats Remberger, Prof. ^3,4^, Ahmed El-Serafi, Ass. Prof.^1,5^, Fadwa Benkessou, MSc.^1^, Wenyi Zheng, MSc.^1^, Eva Martell, researcher^3^, Per Ljungman, Prof.^3^ , Jonas Mattsson, Prof.^3,4^ and Moustapha Hassan, Prof.^1,6^*

^1^ECM, KFC, Department of Laboratory Medicine, Karolinska Institutet, Stockholm, Sweden.

^2^Department of Clinical and Experimental Medicine, Linköping University, Linköping, Sweden.

^3^Center for Allogeneic Stem Cell Transplantation, Karolinska University Hospital, Stockholm, Sweden.

^4^Department of Oncology and Pathology, Karolinska Institutet, Stockholm, Sweden.

^5^College of Medicine, University of Sharjah, UAE.

^6^Experimental Cancer Medicine, Clinical Research Center, Karolinska University Hospital, Huddinge, Sweden.

Email: [ibrahim.el.serafi@ki.se](mailto:ibrahim.el.serafi@ki.se), [mats.remberger@ki.se](mailto:mats.remberger@ki.se), [aelserafy@sharjah.ac.ae](mailto:aelserafy@sharjah.ac.ae), [fadwa.benkessou@ki.se](mailto:fadwa.benkessou@ki.se), [wenyi.zheng@ki.se](mailto:wenyi.zheng@ki.se), [eva.martell@sll.se](mailto:eva.martell@sll.se), [per.ljungman@ki.se](mailto:per.ljungman@ki.se), [Jonas.mattsson@ki.se](mailto:Jonas.mattsson@ki.se), [moustapha.hassan@ki.se](mailto:moustapha.hassan@ki.se)

***Corresponding author:**

Moustapha Hassan, Experimental Cancer Medicine (ECM), Clinical Research Centre (KFC), Department of Laboratory Medicine, Karolinska Institutet Huddinge, Novum, 141 86 Stockholm, Sweden, Telephone: +46-8-585 838 62, Mobile Phone: +46-73-699 88 31, Fax:; +46-8-58583800 email: [moustapha.hassan@ki.se](mailto:moustapha.hassan@ki.se)

**Running title:** NAC effect on liver toxicity during busulphan conditioning


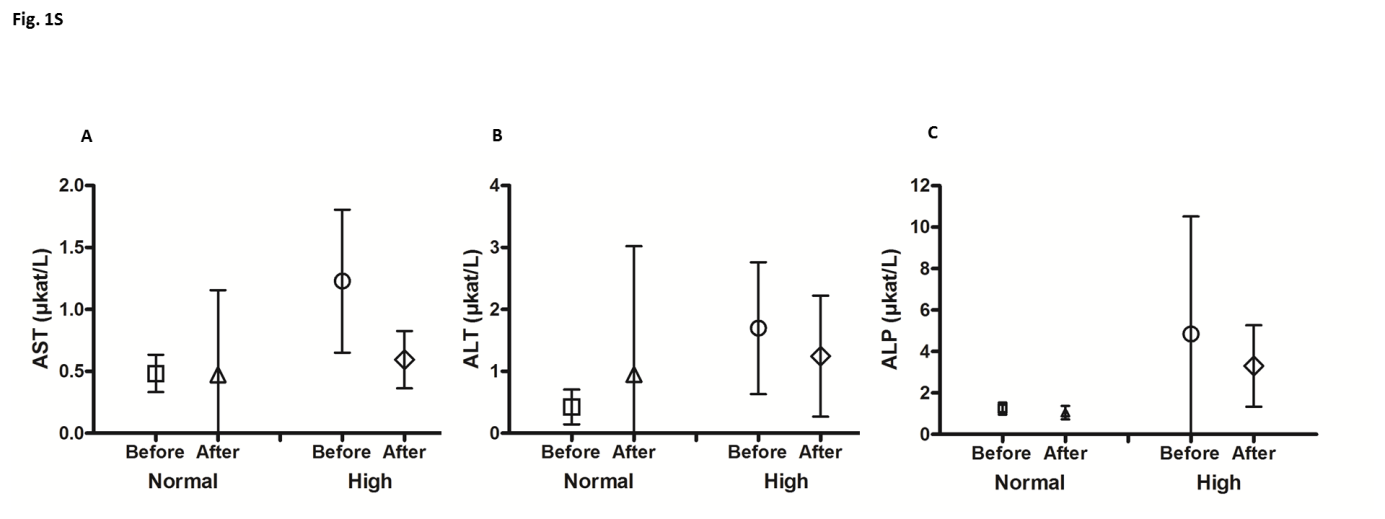


***Fig. 1S. The mean aspartate transaminase (AST), alanine transaminase (ALT) and alkaline phosphatase (ALP) values before and after busulphan conditioning in the control group after subdividing them according to their liver status before the start of the conditioning.***


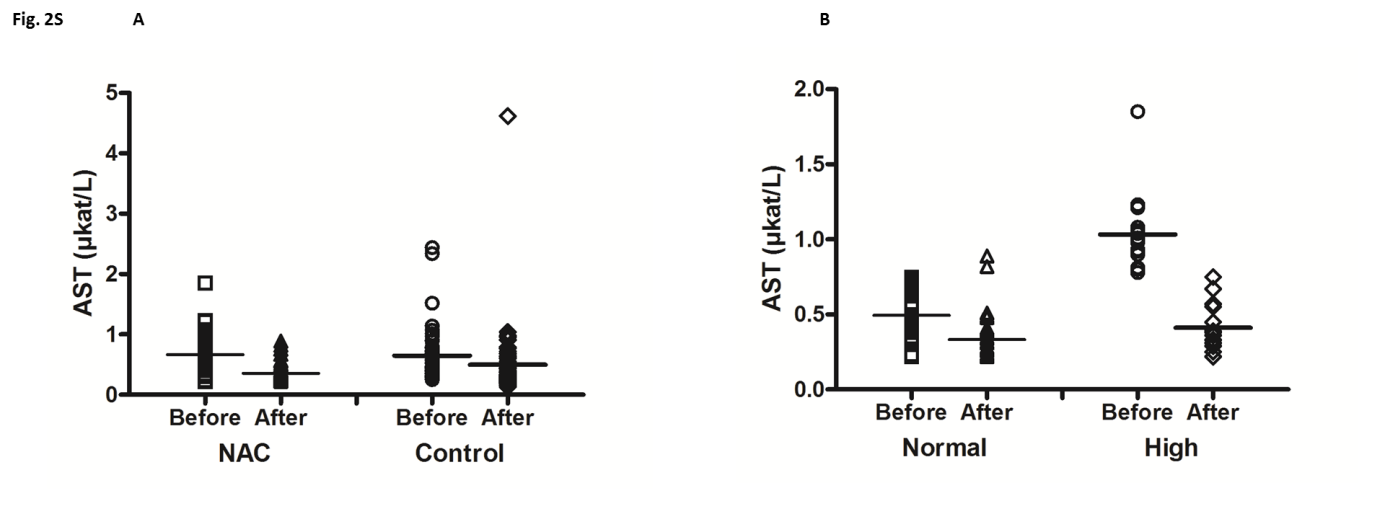


***Fig. 2S. The aspartate transaminase (AST) individual values before and after busulphan conditioning: A) Patients treated with N-acetyl-l-cysteine (NAC) versus the control group, B) Patients treated with NAC and had normal liver values before the start of the conditioning versus patients treated with NAC and had high liver values before the start of the conditioning.***


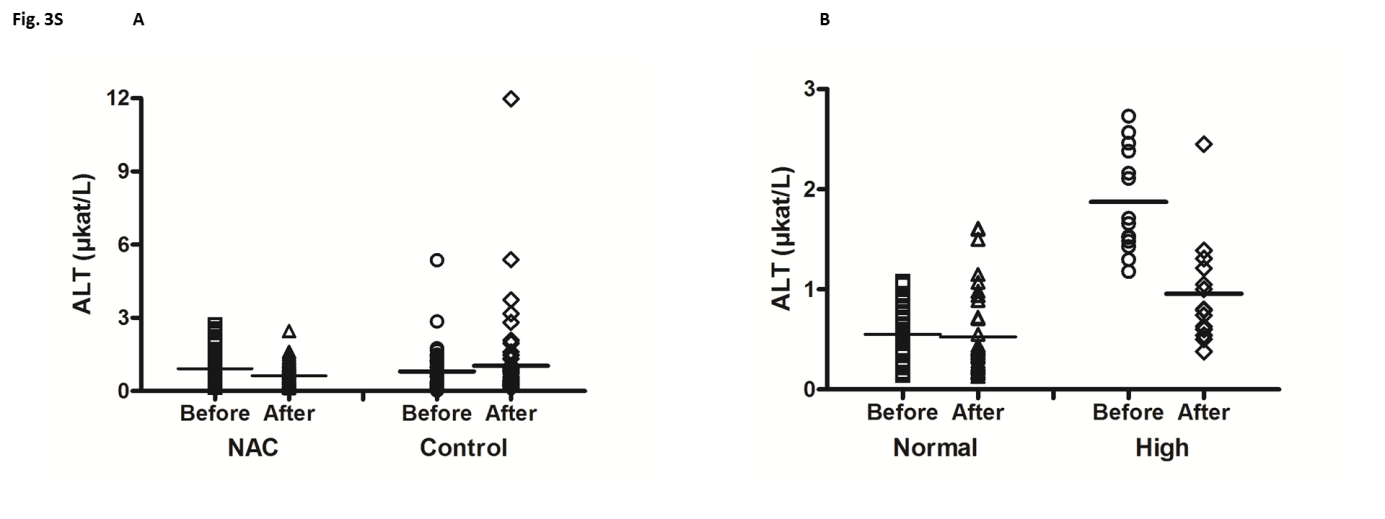


***Fig. 3S. The alanine transaminase (ALT) individual values before and after busulphan conditioning: A) Patients treated with N-acetyl-l-cysteine (NAC) versus the control group, B) Patients treated with NAC and had normal liver values before the start of the conditioning versus patients treated with NAC and had high liver values before the start of the conditioning.***


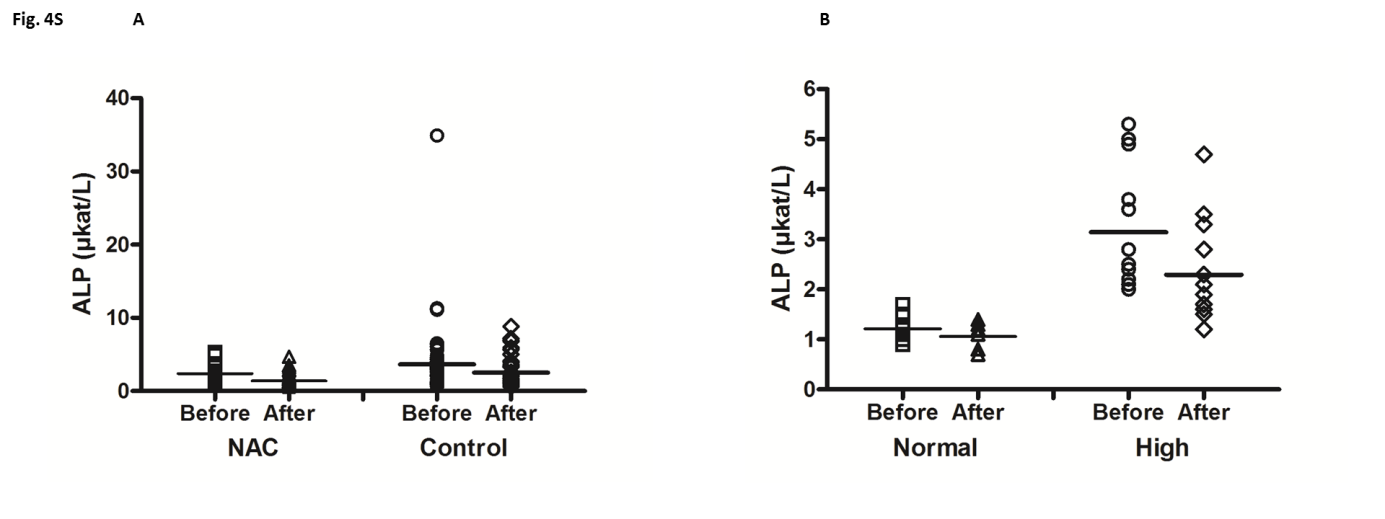


***Fig. 4S. The alkaline phosphatase (ALP) individual values before and after busulphan conditioning: A) Patients treated with N-acetyl-l-cysteine (NAC) versus the control group, B) Patients treated with NAC and had normal liver values before the start of the conditioning versus patients treated with NAC and had high liver values before the start of the conditioning.***


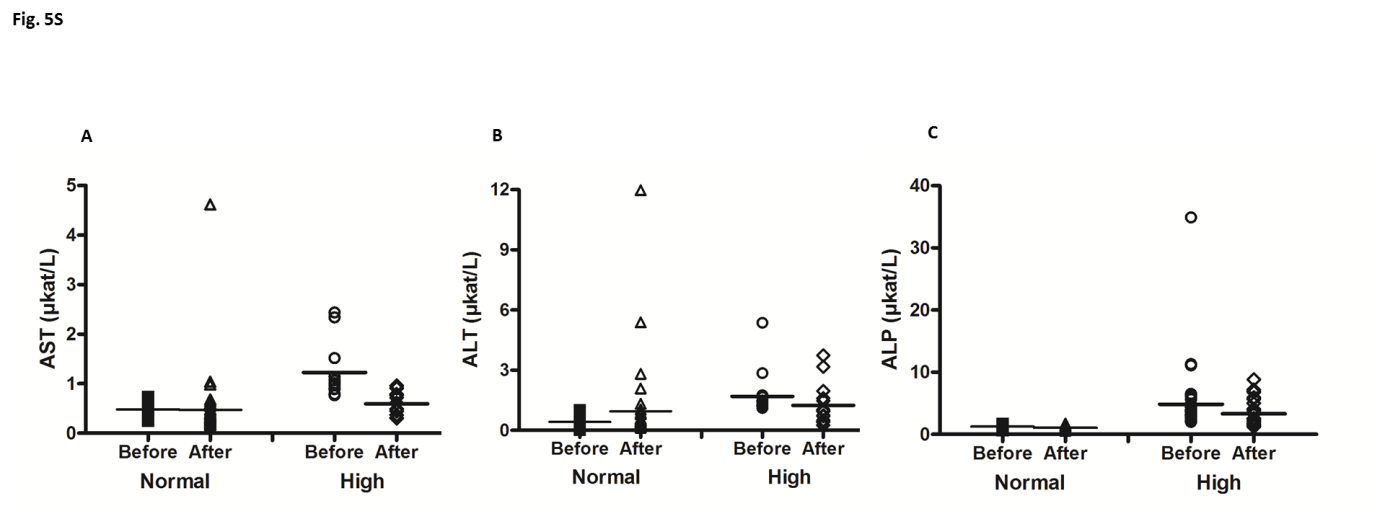


***Fig. 5S. The aspartate transaminase (AST), alanine transaminase (ALT) and alkaline phosphatase (ALP) individual values before and after busulphan conditioning in the control group after subdividing them according to their liver status before the start of the conditioning.***


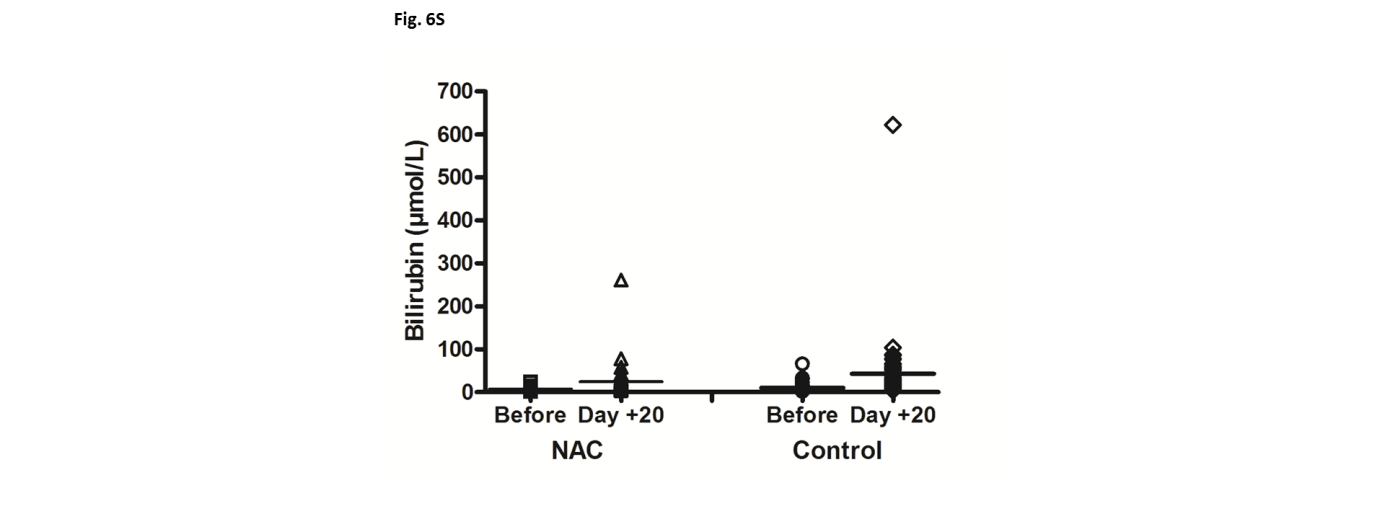


***Fig. 6S. The bilirubin individual values before and after busulphan conditioning in patients treated with N-acetyl-l-cysteine (NAC) versus the control group.***
